# Transient helicity in intrinsically disordered Axin-1 studied by NMR spectroscopy and molecular dynamics simulations

**DOI:** 10.1371/journal.pone.0174337

**Published:** 2017-03-29

**Authors:** Rainer Bomblies, Manuel Patrick Luitz, Sandra Scanu, Tobias Madl, Martin Zacharias

**Affiliations:** 1 Physik-Department, Technische Universität München, Garching, Germany; 2 Department Chemie, Technische Universität München, Garching, Germany; 3 Institute of Structural Biology Helmholtz Zentrum München, Neuherberg, Germany; 4 Institute of Molecular Biology & Biochemistry Center of Molecular Medicine, Medical University of Graz, Graz, Austria; Weizmann Institute of Science, ISRAEL

## Abstract

Many natural proteins are, as a whole or in part, intrinsically disordered. Frequently, such intrinsically disordered regions (IDRs) undergo a transition to a defined and often helical conformation upon binding to partner molecules. The intrinsic propensity of an IDR sequence to fold into a helical conformation already in the absence of a binding partner can have a decisive influence on the binding process and affinity. Using a combination of NMR spectroscopy and molecular dynamics (MD) simulations we have investigated the tendency of regions of Axin-1, an intrinsically disordered scaffolding protein of the WNT signaling pathway, to form helices in segments interacting with binding partners. Secondary chemical shifts from NMR measurements show an increased helical population in these regions. Systematic application of MD advanced sampling approaches on peptide segments of Axin-1 reproduces the experimentally observed tendency and allows insights into the distribution of segment conformations and free energies of helix formation. The results, however, were found to dependent on the force field water model. Recent water models specifically designed for IDRs significantly reduce the predicted helical content and do not improve the agreement with experiment.

## Introduction

The structure–function paradigm of molecular biology, stating that every protein exerting a function requires one specific three-dimensional form, has been under revision since the turn of the century [1]. Evidence has accumulated that some active proteins do not adopt a single stable energy minimum at a folded structure but are intrinsically disordered in solution (intrinsically disordered proteins: IDPs) [2, 3]. Distinct sequence patterns predicted to form intrinsically disordered states in solution have been identified in genome sequences of many forms of life but are more abundant in highly evolved eukaryotes [4, 5]. About 15–45% of eukaryotic proteins have segments of significant disorder [6] where 30 or more consecutive residues are in a disordered state [7, 8]. The relative frequency of IDPs in the more communicative eukaryotes compared to prokaryotes is reflected in their important role in transcription, translation, cell cycle regulation and cell signaling [9–13]. Analysis of the SwissProt data bank revealed that many diseases, including cancer, malaria, human immunodeficiency virus (HIV) and acquired immunodeficiency syndrome (AIDS), deafness, obesity, cardiovascular diseases, diabetes mellitus, albinism, and prion protein related diseases, are correlated with proteins predicted to contain disordered regions [14].

Several experimental techniques can be used to qualitatively characterize the disordered nature of proteins [6]. Obtaining information on the ensemble of disordered states or transiently formed structures is, however, experimentally difficult, as typically only averages over time or large sample sizes can be evaluated. Also IDP systems tend to be underdetermined, i.e. the number of experimental observables is below the number of conformational states. Computational studies are, in principle, ideally suited for the study of IDPs, allowing single molecule investigation with a spatial resolution up to single atoms and a time resolution of femtoseconds [15]. Especially in combination with experimental techniques like nuclear magnetic resonance (NMR) spectroscopy an atomistic understanding of the range of accessible conformations is available [16, 17].

Molecular dynamics(MD) simulations model protein behavior by propagating all atom positions according to Newton’s equations of motion [18] and estimating forces on atoms via an empirical force field. Current force fields describe folded globular proteins well [19], but overstabilize protein–protein interactions [20] and often fail to reproduce realistic IDP behavior [21–23]. Most force fields succeed in predicting qualitatively whether or not a region is disordered, but for further details, like dynamics or sampled sub-states, results vastly differ between different force fields [23]. Two groups have approached this problem by parameterizing new water models. Both approaches tackle the overpopulation of collapsed states with current force fields by modifying the solute–solvent interactions.

Best et al. [21] argue that pure water properties are reproduced well by current water models and thus water–water interactions should not be altered. For their adjusted water, termed amber03ws, the strength of solvent–solute van der Waals interactions is increased by a factor of *γ* = 1.1, obtained from fits to the temperature profile of Förster resonance energy transfer (FRET) data for the Csp M34 protein. Extensive tests of the new force field included comparison with Small-Angle X-ray Scattering (SAXS) data, solvation free energies of amino acid analogues, protein self-association, the intrinsic structure propensity in short peptides, the helix-coil transition, the folding of mini-proteins and the stability of folded proteins.

According to another approach by Piana et al. [22], the dispersion component of the intermolecular interaction energy is underestimated in current water models. They fitted the *C*_6_ term of Lennard-Jones interactions to quantum level computations and adjusted partial charges and *C*_12_ to fit density and vaporization enthalpy temperature profiles of their TIP4P-D water model. The water model was tested in a total simulation time of 830μs by checking the solvation free energies of side chains, radii of gyration of disordered proteins and a comparison with SAXS, FRET and NMR paramagnetic relaxation enhancement data for *α*-Synuclein. Both water models performed well in test simulations and in particular sampled larger radii of gyration in agreement with experiments.

Aside from radii of gyration MD force fields have to reproduce very diverse properties of disordered regions. Often, disordered regions in proteins are involved in the interaction with other biological binding partners and can fold upon binding adopting different conformations. Among these, the *α*-helical structure is the most abundant secondary structure. Hence, in practice one is interested in the ability of force field simulations to distinguish segments from IDPs that have an intrinsic preference for adopting helical secondary structure and can bind involving a conformational selection mechanism or prefer fully disordered states. Here we use the intrinsically disordered, transiently folded Axin-1 protein as model system to compare computational approaches for conformational characterization of IDPs.

Axin-1 is a key protein component of the Wnt signaling pathway [24] acting as a scaffolding protein, assembling the β-catenin destruction complex that phosphorylates and subsequently polyubiquitinates β-catenin [25–27]. The central region of the 862 residue protein Axin-1 (residues 212-780) is highly susceptible to proteolytic degradation [28] and has been proposed to be largely intrinsically disordered [29]. Nevertheless, the disordered region is essential for β-catenin trapping and subsequent degradation in the β–catenin destruction complex. For these purposes, the disordered region of Axin-1 harbors binding sites for β-catenin (residues 466-480) and the kinases Glycogen synthase kinase 3β (GSK3β; residues 383-400), and Casein Kinase 1 (CK1) [30–32]. In complex with each of the binding partners, Axin-1 adopts a helical structure in the respective binding region [30, 32]. The intermediate region connecting the binding sites (residues 430-450) is proline-rich and predicted to be in a coiled coil state [33].

In the present study we investigate the helix propensity of the Axin-1 residues 380-490 by segmenting the amino acid sequence in peptides of 10 residues and assessing the conformational space of these peptides with an advanced sampling MD simulation method. Simulation results are directly compared to results obtained from NMR spectroscopy on the Axin-1 segment. Good qualitative agreement between MD simulations and experiment is found, indicating that segments that bind to signal proteins indeed adopt already partially helical conformations in the absence of the binding partner. However, significant differences between force field description and type of water model used in the simulations are found. While the amber99SBws [21] force field with corrections to the backbone parameters reproduces enhanced helicity in certain sequences, TIP4P-D [22] water does not reproduce transient helical population.

## Materials and methods

### Simulation of the helical propensity

The relevant section of Axin-1 (residues 380-490: Axin-1_380−490_) is, at least for current computer power, too large to be simulated for timescales on which the complete conformational space could be assessed. In order to obtain an estimate of helicity in different areas of Axin-1_380−490_, we split the amino acid sequence into segments of 10 residues. Sequences of these segments are indicated in the Supporting Information, Table A in S1 File. For each of these peptides we ran an advanced sampling MD simulation protocol to estimate the free energy (or potential of mean force, PMF) along a reaction coordinate that maps the helicity of the peptide. As reaction coordinate we used the root mean square deviation of a set of *i* distances (dRMSD) from a respective set of *i* reference values:

$$R\left(d_{1},\ldots ,d_{N}\right)=\sqrt{\frac{1}{N}\sum_{i}^{N}{\left(d_{i}-d_{i,0}\right)}^{2}}$$ (1)

The dRMSD coordinate *R* defines a structure using a set of characteristic reference distances *d*_*i*,0_. During the simulation the actual distances *d*_*i*_ are compared to the reference distances and an average deviation termed *R* is defined (see Eq 1). In the present case, with the helical reference state all distances between *C*_*α*_ atoms *C*_*α*,*j*_ − *C*_*α*,*j*+3_ were included, with a reference distance *d*_*i*,0_ = 0.5 nm for every *i*, *R* = 0 represents the fully helical peptide and an increase of the dRMSD coordinate *R* shows increasing deviation from the helical structure. A quadratic potential was employed to limit the sampling to specific regions of *R* around a reference value *R*_0_. The potentials with a force constant *k*_0_ are of the form:

$$V\left(d_{1},\ldots ,d_{N}\right)=\frac{1}{2}k_{0}{\left(R\left(d_{1},\ldots ,d_{N}\right)-R_{0}\right)}^{2}$$ (2)

More details on the reaction coordinate can be found in Supporting Information Description in S1 File.

### Force fields

We used the amber99sb*-ILDN force field for the peptides, which is an revised version of amber99sb [34] with improved side chain torsion parameters [35] optimized for helix-coil transitions [36]. For the solvent the classical TIP3P [37] water was compared to new force fields explicitly developed for intrinsically disordered proteins. TIP4P-ws [21] comes with an increased *k*_Ψ_ for the protein backbone from 0.75 kJ/mol to 2.0 kJ/mol, as indicated in the respective SI [21]. To compare solely the performance of the water force field we also tested a variant we termed TIP4P-s that does not edit the protein force field by adjusting *k*_Ψ_. The set of tested water force fields was completed with TIP4P-D [22].

### Simulation protocol

All simulations were conducted using GROMACS 4.6.5 [38], applying periodic boundary conditions and covering long-range electrostatic interactions with the Particle-Mesh-Ewald [39] method with a Fourier-spacing of 0.16 nm and a grid interpolation up to order 4. Close Van-der-Waals and Coulomb interactions were cut off at a radius of 1.0 nm. Long range dispersion correction was applied to account for errors from truncated Lennard-Jones interactions. Bond lengths of H-atoms were constrained with the LINCS [40] algorithm and a coupling matrix extension order of 4 (12 in equilibration runs). All systems were run with a step size of 2 fs at a temperature of 300 K, controlled by velocity rescaling [41], and a pressure of 1.01 bar with the Parrinello-Rahman barostat [42].

Starting structures of the peptides were generated with PyMol [43] in a helical conformation and with ACE and NH2 caps at the ends. The peptides were solvated and in a dodecahedral box large enough to accommodate the fully unfolded molecules and containing, depending on the specific sequence, approximately 2000 water molecules. Initial energy minimization with the steepest descent algorithm was stopped when the maximum force dropped below 100.0 kJ/mol/nm or after 25000 steps. Subsequent equilibration was performed per replica with a time step of 1 fs and the velocity rescaling thermostat [41] for 50.000 steps as NVT equilibration and for 100.000 steps with the Berendsen barostat [44] as NPT equilibration.

For each segment the free energy landscape was sampled in 12 equally spaced *λ*-windows for dRMSD values between 0.0—0.5 nm. Replica exchange between windows was attempted every 500 steps to further enhance convergence and overcome potential artificial energy barriers.

### Protein preparation

The DNA construct containing the Glu390-Val500 region of human Axin-1 (uniprot reference O15169) was purchased from ATG:biosynthetics GmbH within a pUC cloning vector. The DNA sequence was codon optimized for protein production in bacterial cells and flanked by NcoI and BamHI restriction sites. The coding region was cloned into a modified pETM-11 bacterial expression vector (kindly provided by Arie Geerlof Protein Expression and Purification Facility, Helmholtz Zentrum München, Germany) which was derived from a pET-24d(+) vector (Novagen) by insertion of a tobacco etch virus (TEV) protease cleavage site following a N-terminal hexa-histidine and a protein A tag. Axin-1 gene was amplified by PCR using T4 primers (New England Biolabs). The resulting PCR products and pETM-11 were double digested with NcoI and BamHI enzymes (New England Biolabs) before ligation. The construct was verified by sequencing. The numbering of Axin-1 amino acid residues follows the full length protein as reported on the UniProt website. Uniformly (^13^C,^15^N) double-labelled protein was produced in freshly transformed *E.coli* DE3 cells. A single colony was inoculated in Luria-Bertani medium (20ml) with kanamycin (25mg/l) and cultured at 37°C until the OD_600_ reached a value between 2 and 3. From this, an aliquot (1ml) was added to (^13^C,^15^N labelled) M9 minimal medium (100ml) in which ^15^N-NH_4_Cl (1g/l) and ^13^C-glucose (2g/l) were the only sources of nitrogen and carbon for NMR isotope labelling purposes (Cambridge Isotope Laboratories, Inc). The culture was incubated overnight at 37°C and shaken at 180rpm. Fresh (^13^C,^15^N) M9 minimal medium was added up to 1l, and the culture was grown under the same conditions until the OD_600_ reached 0.8. Protein expression was induced with 1mM β-D-1-thiogalactopyranoside (IPTG) at 18°C. The cells were pelleted next day by centrifugation using a Fiberlite F9-6x1000 rotor in a Sorvall LYNX 6000 Superspeed centrifuge at 2000g for 20 minutes. Re-suspension and contemporaneous cell lysis were obtained adding 40mL of 50mM NaPi, 300 mM NaCl, 8 M urea, pH 8 and gentle stirring at room temperature for 20 minutes. The cell lysate was separated by ultracentrifugation using a Thermo Scientific SS-34 rotor in a Sigma 6K 15 centrifuge at 20,000 g for 30 min at 4°C and histidine-tagged protein was affinity-purified via Ni-NTA resin (Qiagen). TEV protease (5 μg/ml) was added to the eluate and dialysed overnight at 4°C against 50 mM Tris(hydroxymethyl)amminomethane chloride (TRIS-HCl), 150 mM NaCl, 0.5 mM tris(2-carboxyethyl)phosphine (TCEP), 2mM β-mercaptoethanol, pH 7.5. The dialysed solution underwent heat shocking at 90°C for 30 minutes, followed by ultracentrifugation using the Thermo Scientific SS-34 rotor in Sigma 6K 15 centrifuge at 20,000g for 30 min at 4°C. Axin-1 was separated from the tag by a size-exclusion chromatography step, using a Superdex 75 10/300 GL column (GE Healthcare), equilibrated with 20 mM NaPi, 300 mM NaCl, 2 mM 1,4 Dithiothreitol (DTT), pH 6.5, on an ÄKTA pure FLPC system. The concentration of the protein was estimated by absorption spectroscopy (*ϵ*_280_ = 5.6 mM^−1^cm^−1^). The purity was estimated by SDS-PAGE to be 95% with a yield of pure protein being 10 mg per litre of culture.

### NMR spectroscopy

Axin-1 (residues 390-500) sample (0.5 mM) with 0% and 40% TFE contained 20 mM NaPi, 300 mM NaCl, 2 mM DTT, pH 6.5 and 10% D_2_O for lock. Triple resonance backbone assignment experiments included: CBCA(CO)NH, CBCANH, HNCA, HNCAN(N)H, HNCANN(H), HCCC(CO)NH and HCCH-TOCSY. All NMR spectra were recorded at 298 K on Avance III 600 MHz and Avance II 900 MHz Bruker spectrometers, equipped with TCI (^1^H, ^13^C, ^15^N) with z-gradient and TXI (^1^H, ^13^C, ^15^N) with z- and xyz-gradient cryoprobes, respectively. Spectra were processed with NMRPipe [45] and analyzed with CcpNMR Analysis [46]. The secondary structure propensity (SSP) was derived by the online tool ncSPC [47] (neighbour-corrected Structural Propensity Calculator), according to

$$\text{SSP}=\Delta \delta C_{\alpha }-\Delta \delta C_{\beta }$$ (3)

where Δ*δC*_*α*_ and Δ*δC*_*β*_ represent the difference between carbon *α* and carbon *β* Axin-1 experimentally obtained chemical shifts and carbon *α* and carbon *β* random-coil chemical shift references, respectively.

## Results

### Free energy landscape along the amino acid sequence

**Fig 1:**
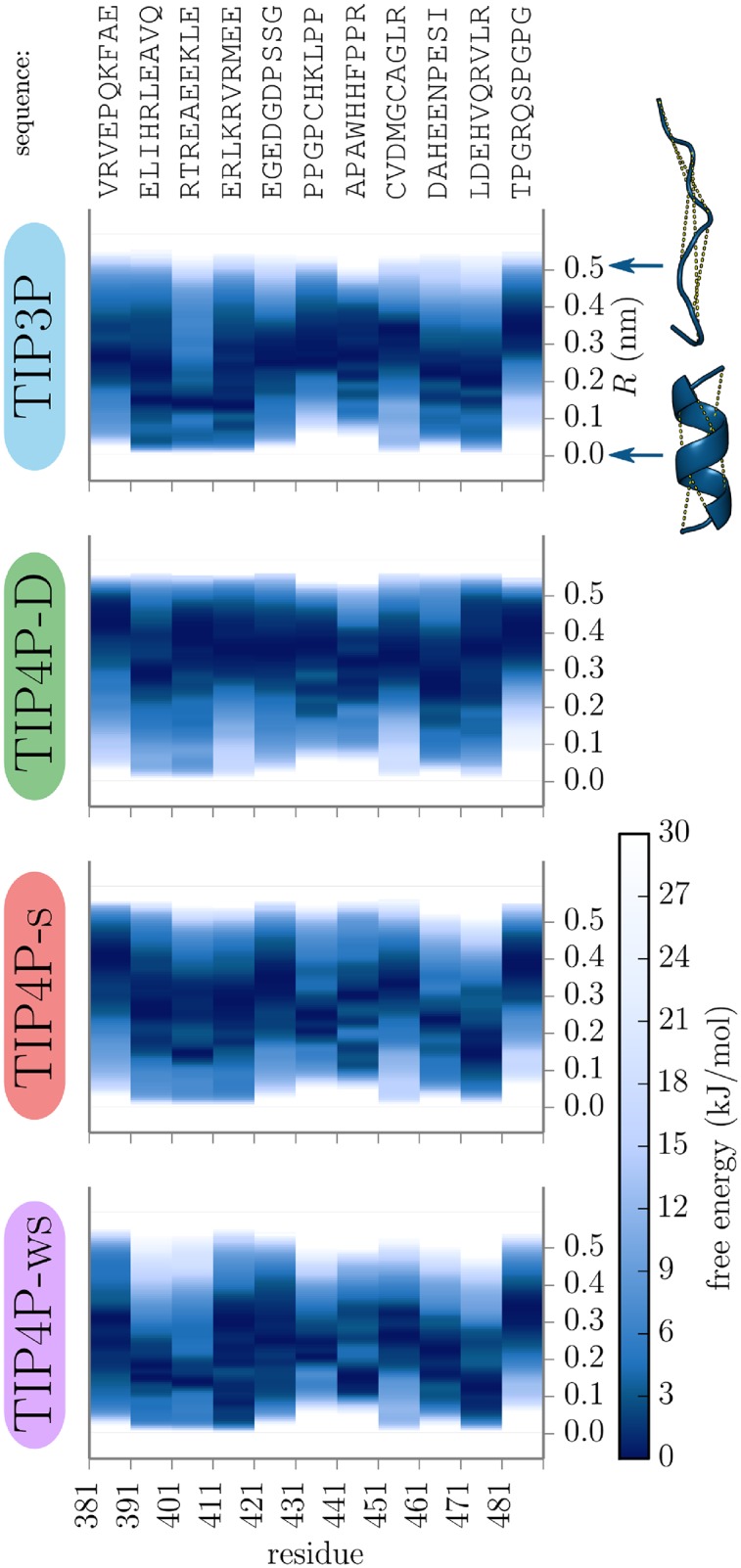
Free energy of unfolding for segments of Axin-1_380−490_. Free energy landscape for segments of Axin-1_380−490_ along the unfolding reaction coordinate *R* with different water force fields.

The free energy landscape of helix formation for the 11 segments of Axin-1_380−490_ with four tested water models is shown in Fig 1. With TIP3P water the regions of *R* with the lowest free energy fluctuate from segment to segment. While some segments, residues 390-420, 440-450 and 460-480, have their global minimum close to the helical structure at *R* < 0.2, the other segments prefer unfolded structures with the global minimum around *R* ∼ 0.3 and above. With TIP4P-D almost all segments show global minima at very high *R* ∼ 0.4–0.5 where the peptide chain is almost fully extended. Shifts of the global free energy minimum in the *R*-axis between segments are reduced and helical states with small *R*-values are generally disfavored. The free energy landscape with TIP4P-s favors or disfavors helical structures for the similar ranges of residues as TIP3P. TIP4P-ws with a backbone correction towards more helical states indeed shifts the minima towards lower values of *R*.

### Population of a helical state

For *in silico* studies, the definition of a helical state of a peptide is not straight-forward. At atomic resolution helicity is often classified using the DSSP algorithm [48], based on an energy function for the backbone hydrogen bond stabilizing the helix. Overall helicity decreases linearly with *R*. For *R* = 0.15 the DSSP helicity drops below 50%. This value of *R* was subsequently used as an upper boundary for the helical state of a peptide. The average DSSP helicity of all residues of all peptide sequences is plotted with respect to the dRMSD *R* in the Supporting Information, Fig A in S1 File.

**Fig 2:**
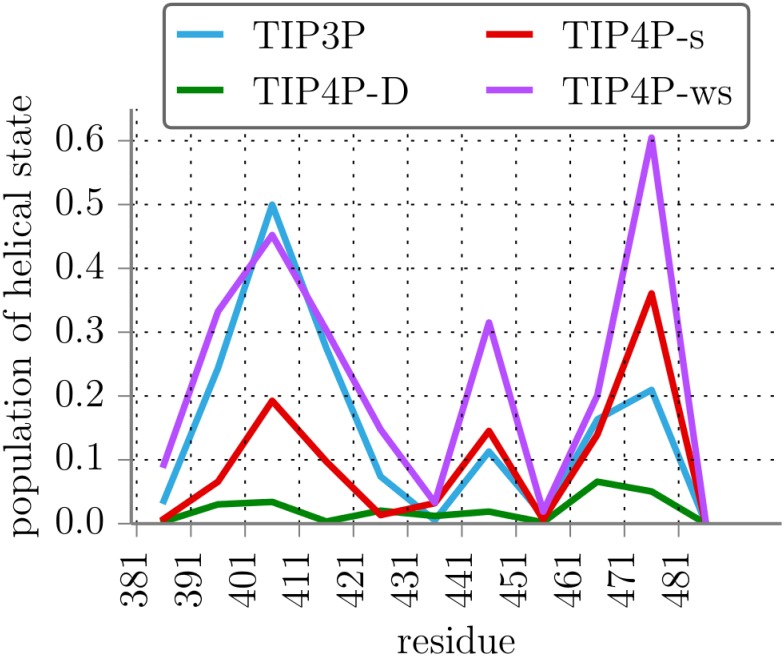
Helicity of Axin-1_380−490_ segments. Fraction of population of a helical state for a range of segments of Axin-1_380−490_ with different water force fields.

Population of this helical state is displayed in Fig 2 for each segment. TIP3P predicts transiently helical segments between residue numbers 390 and 420, a small helical population between residues 440 and 450 and further transient helicity between residues 460 and 480. With TIP4P-s the same residue regions show a relevant population of the helical state, but the 390-420 region is less helical while in the 460-480 region especially the segment starting at residue 471 is more helical than with TIP3P. Simulations with TIP4P-ws reproduce and amplify the helicity peaks of those with TIP4P-s. Finally, with TIP4P-D almost no increase of helical populations in the respective regions can be seen. Note that the position of the helical boundary at *R* = 0.15 nm(see Supporting Information, Fig A in S1 File) affects the absolute values of the population, but the relations between segments and force fields persist.

### Helix population from NMR secondary chemical shifts

**Fig 3:**
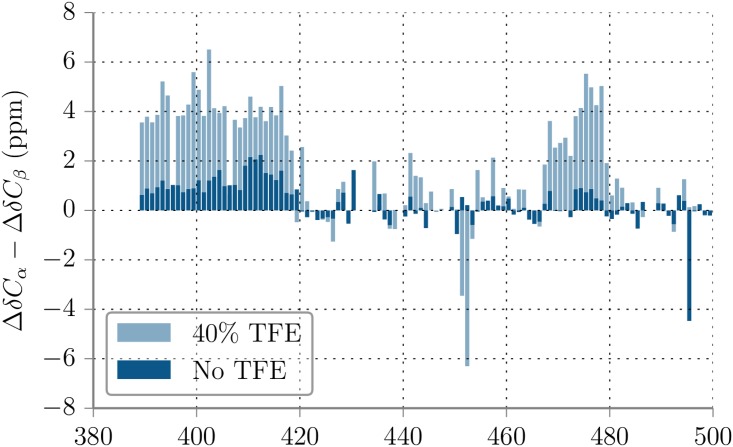
Experimental secondary structure propensity. Secondary structure propensity of Axin-1 (residues 390-500) in 40%TFE and without TFE, obtained from NMR chemical shift measurements. Consecutive positive values indicate *α*-helices, negative values indicate β-sheets.

To evaluate the population of secondary structure experimentally, NMR spectroscopy was used. In line with previous work [29], two-dimensional ^1^H–^15^N heteronuclear single-quantum coherence (HSQC) NMR correlation spectra recorded on isotope-labeled Axin-1 protein (residues 390-500) showed that the protein is indeed largely disordered as indicated by clustering of resonances in a narrow range of 7.6—8.6 ppm (Fig 3). Nevertheless, NMR-derived secondary chemical shifts indicate that both the GSK3β and β-catenin binding sites adopt transient *α*-helical conformation. In line with this, addition of TFE, an agent stabilizing *α*-helical structure [49], increased the population of *α*-helical conformation (Fig 3). Based on the secondary NMR chemical shifts obtained in presence of TFE, Axin-1 adopts a helicity of approx. 40% for residues 390-420 and 15% for residues 470-480 in the native state (meaning in the absence of TFE), respectively. Comparison with the simulation results indicates that the simulations with TIP3P water give best agreement with the NMR results both in overall magnitude of the predicted residual helicity but also in the relative helicity of the two segments (only for the TIP3P model a higher helicity of the residues 390-420 compared to 470-480 is found, compare Figs 2 and 3).

### Differences between force fields

**Fig 4:**
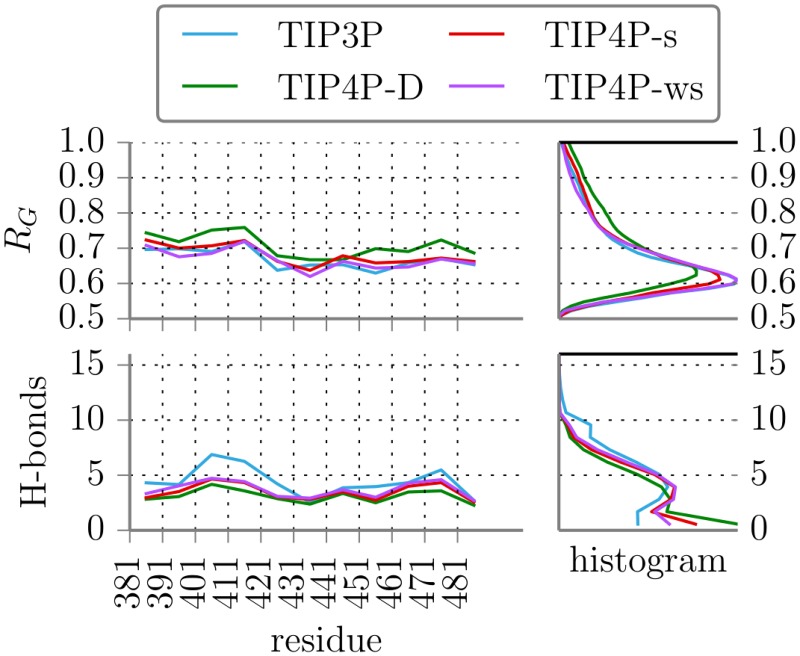
Radii of gyration and numbers of H-bonds. Averages per segment and total histograms of radius of gyration and number of intra-molecular H-bonds of the peptides for different force fields.

The simulation results on the peptides using the four water models also indicated differences in the sampled peptide structural properties. Fig 4 shows the averages per segment and global histograms of the radius of gyration and number of intra-molecular H-bonds of the peptides in different force fields. In the simulations with TIP4P-D states with a larger radius of gyration were consistently sampled more frequently than with the other water force fields. With TIP3P, instead, more states with a higher number of intra-molecular hydrogen bonds were sampled. Especially for the segments 401-410 and 411-420 many intra-molecular H-bonds persisted. In the segment starting at residue 401 two Arg-Glu pairs stabilize the helical fold: Arg403-Glu410 and Arg401-Glu404 on opposite sides of the helix (snapshots of structures in Supporting Information, Fig B in S1 File). With TIP3P water these two bonds are more persistent than with TIP4P-ws and far more persistent than with TIP4P-D. Similarly, a stable bond between Arg412-Glu420 stabilizes a slightly kinked helical motif and is sampled the least by TIP4P-D.

**Fig 5:**
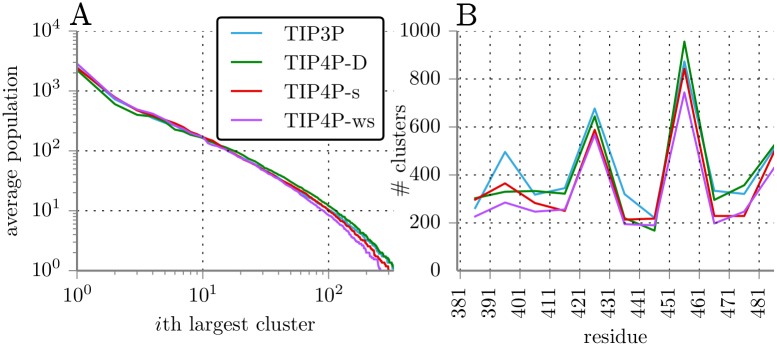
Cluster sizes for the different force fields. (A) Average size of clusters after sorting clusters by size for each force field. (B) Number of populated clusters for each segment and force field.

To find further differences in the sampled structures we clustered the trajectories of all replicas and force fields with the method of Daura et al. [50]. This can provide information on the degree of diversity of sampled structures and can tell whether or not some structures are strongly favored only for a specific force field. Fig 5 shows average cluster sizes after sorting the clusters by size for each force field. A tendency of TIP4P-D can be observed to sample highly populated clusters less and sparsely populated clusters more. In a second plot Fig 5 furthermore shows the number of populated clusters for each force field and segment. All tested water force fields sampled similar cluster size distributions. The numbers of clusters in the different segments approximately agree for all force fields. Only for the segment starting at residue 391 simulations with TIP3P sample a larger number of clusters than the modified IDP water force fields. Simulations with both TIP4P-ws and TIP4P-s systematically sampled fewer clusters than the other two.

**Fig 6:**
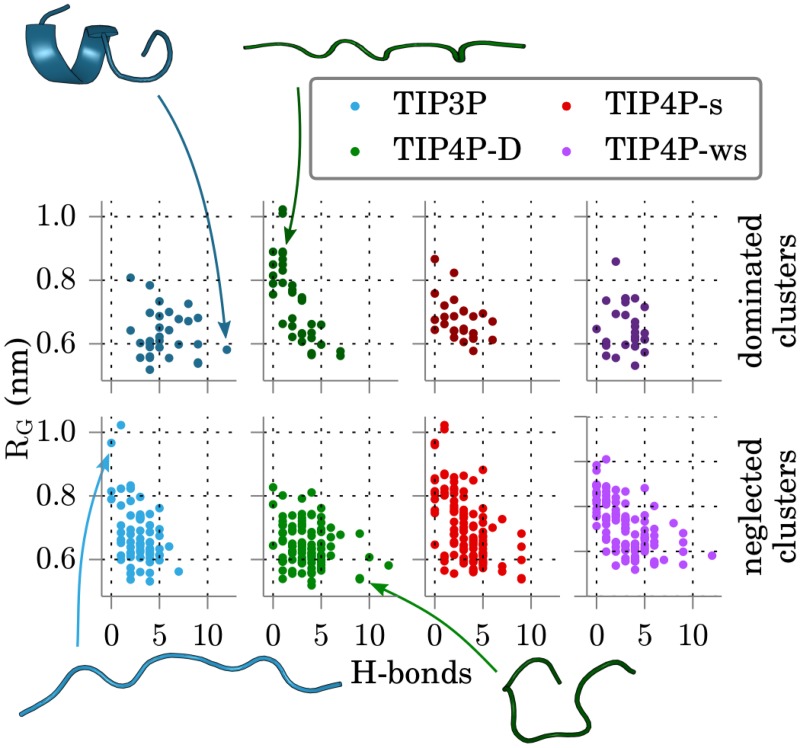
Properties of dominated and neglected clusters. Radii of gyration and number of intra-molecular H-bonds for all clusters dominated or neglected by the different water force fields. Clusters were considered dominated by a force field when more than 80% and neglected if less than 1% of their structures was obtained with that force field. Representatives of indicated clusters are shown as cartoon.

A more detailed look at the obtained clusters involved checking for structures predominantly sampled by specific force fields. We identified all significant clusters (i.e. containing more than 100 structures) of all segments that were dominated or neglected by a force field. Clusters were considered neglected if less than 1% and dominated if more than 80% of the cluster structures were contributed from simulations with one force field. We found 118 dominated clusters (30 TIP3P, 35 TIP4P-D, 27 TIP4P-s, 26 TIP4P-ws) and 378 neglected clusters (68 TIP3P, 93 TIP4P-D, 108 TIP4P-s, 109 TIP4P-ws). In Fig 6 the radius of gyration and the number of intra-molecular H-bonds of all such dominated or neglected clusters are plotted. TIP3P dominates for structures with small *R*_*G*_ but with many H-bonds and neglects structures with larger *R*_*G*_. TIP4P-D dominates structures with large radii of gyration and neglects several clusters with many H-bonds. TIP4P-s and TIP4P-ws both dominate fewer clusters than the first two water models and neglect more clusters. Dominated clusters are all in the small *R*_*G*_ and modest number of H-bonds regime, and neglected clusters include extended peptides, more than TIP3P, as well as structures with many H-bonds.

### Convergence

To test convergence of the obtained PMFs we seperated the data in four equal sets(see Supporting Information, Fig D in S1 File). Most PMFs for datasets after the first quarter do not shift any more. Some segments, notably starting at residue 431 and 441, still show a continued decrease of the PMF in the unfolded region, but the overall shape of the free energy remains similar. Obtained PMFs from the four different force fields show very similar shapes for each segment except for the precise preference of helical states and thus are considered converged. The similar numbers of sampled clusters are a further indication that (at least a comparable level of) convergence has been reached in all simulations.

## Discussion

NMR secondary chemical shifts and MD simulations provided evidence that the central Axin-1 segment is largely disordered but shows areas of helix propensity, especially in the binding regions of GSK-3β and β-catenin. The transiently folded regions identified here agree with results from the neural network predictor PONDR [33], where residues approximately in the ranges 380 − 400 and 450 − 480 show a reduced disorder score. Segmenting the 100 amino acid chain into peptides of 10 residues we were also able to identify regions of increased helicity in MD simulations. The used water model, however, has a significant impact on the structural ensemble of 10 amino acid peptides.

The simulations employing the TIP3P water model reproduce the higher helix propensity for the binding regions, as observed in the NMR experiments. The helix propensity is in the same order as the experimentally observed helix formation probability. In addition, the relative helicity of the two binding regions as observed in the NMR experiment was correctly predicted when using the TIP3P water model. Newer water models, specifically adapted to describe IDPs, reduce the helicity of all segments. TIP4P-s reduces overall helicities, but differences between segments persist and helical regions are still identified. With the backbone correction of TIP4P-ws absolute helicities of TIP3P are reproduced or even exceeded. With TIP4P-D water, folded states are energetically penalized and all peptides strongly favor unfolded conformations. In particular, the force field tends to underestimate the formation and persistence of secondary structure elements and seems to destabilize salt bridges of side chains, as seen for the Arg-Glu pairs of segments 401 and 411.

The difference in the radii of gyration in Fig 4 seems small but is of importance, as the systems were forced to cover all areas of the dRMSD reaction coordinate, which strongly correlates with *R*_*G*_. Since the sampling is forced to all regions of *R*_*G*_, TIP4P-D must consequently favor larger *R*_*G*_. The same argument goes for the number of H-bonds. At low *R* peptides are forced into completely helical structures already featuring 7 H-Bonds, so all force fields do sample structures with a high count of H-bonds. Yet only TIP3P samples collapsed structures with an even higher number of hydrogen bonds to a relevant degree.

Best et al. [21] tested their new water model with the amber03w protein force field, but in the supplement provided an adaption for amber99sb*-ILDN used in this work. The adapted amber99sb*-ILDN with TIP4P-ws overstabilized helices at higher temperatures in a Ac-(AAQAA)_3_-NH2 peptide, but correctly sampled helix propensities at 300 K. This is in agreement with our simulations, where TIP4P-s, with increased solute–solvent interactions but no adjustment of the protein backbone dihedral potentials, undersampled helical states, but TIP4P-ws with the backbone modifications increased helix propensity. Piana et al. [22] in their validations mostly used the amber99sb-ILDN force field. Their validation results should be valid with amber99sb*-ILDN used here, which only adds a modification to actually improve helix–coil transitions [36]. An explicit test of the helix–coil equilibrium of short sequences was, however, not part of the original force field validation.

## Conclusion

In this work we investigated the property of intrinsically disordered regions to contain transient helical population with MD simulations and NMR secondary chemical shifts. Our model system Axin-1_380−490_ showed intrinsically disordered behavior but increased transient helical content in the binding regions of two binding partners. This result is of significant importance for understanding the function of IDP regions in proteins. Even a small preference for adopting conformations close to the bound structure can significantly modulate the binding capacity of a protein segment. This could be a general basis for fine tuning the binding properties of IDP containing proteins. Interestingly, the predicted degree of residual helicity depended significantly on the selected water force field model. Simulations with the traditional TIP3P water model reproduced the trend of increased helicity in the binding regions quite well. However, water models explicitly parametrized for IDPs underestimated the helical content. In particular, TIP4P-D strongly disfavors collapsed, folded peptide conformations. With corrections to backbone parameters amber99SBws simulations using the TIP4P-ws were able to reproduce and even slightly overestimate the higher helical propensities of the binding regions. Hence, this model or the traditional TIP3P appear to be most appropriate for the present purpose of identifying residual helical structures in IDP segments. Our study indicates that the choice of the force field water model remains to be of critical importance for studying the properties of intrinsically disordered proteins.

## Supporting information

S1 File **Description in S1 File,** The supporting information gives a detailed explanation of the dRMSD reaction coordinate and its implementation in GROMACS [38] and contains the following additional figures and a table. **Table A in S1 File, Sequences of simulated segments of Axin-1**_380−490_. **Fig A in S1 File, Helicity with respect to the dRMSD.** DSSP Helicity of peptides with increasing *R* for different water force fields. The average is taken over all segments and all replicas for each force field. **Fig B in S1 File, Snapshots of diverging typical segment conformations.** Snapshots from the lowest *R* replica of segment 401. A shows two stable double-H-bonds between Arg401-Glu404 and Arg403-Glu410 typically sampled with TIP3P water. B shows a typical snapshot from TIP4P-D water where neither contact is formed. **Fig C in S1 File, Time evolution of PMFs.** Time evolution of all PMFs for all water force fields. Simulation data was evaluated after 10ns of equilibration. Global shapes of the PMFs barely change after the first quarter of evaluated simulation time. **Fig D in S1 File, Schematic of dRMSD PMF.** Schematic depiction of the enhanced sampling with the dRMSD method.
(PDF)
