# Supplementary material for: Transient helicity in intrinsically disordered Axin-1 studied by NMR spectroscopy and molecular dynamics simulations
Source: PLoS One. 2017 Mar 29;12(3):e0174337. doi: 10.1371/journal.pone.0174337 (PMC5371316; doi:10.1371/journal.pone.0174337)
Supplement: S1 File — Description in S1 File, The supporting information gives a detailed explanation of the dRMSD reaction coordinate and its implementation in GROMACS [38] and contains the following additional figures and a table. Table A in S1 File, Sequences of simulated segments of Axin-1380−490. Fig A in S1 File, Helicity with respect to the dRMSD. DSSP Helicity of peptides with increasing R for different water force fields. The average is taken over all segments and all replicas for each force field. Fig B in S1 File, Snapshots of diverging typical segment conformations. Snapshots from the lowest R replica of segment 401. A shows two stable double-H-bonds between Arg401-Glu404 and Arg403-Glu410 typically sampled with TIP3P water. B shows a typical snapshot from TIP4P-D water where neither contact is formed. Fig C in S1 File, Time evolution of PMFs. Time evolution of all PMFs for all water force fields. Simulation data was evaluated after 10ns of equilibration. Global shapes of the PMFs barely change after the first quarter of evaluated simulation time. Fig D in S1 File, Schematic of dRMSD PMF. Schematic depiction of the enhanced sampling with the dRMSD method. (PDF) [file pone.0174337.s001.pdf]

Supporting Information: Transient Helicity in Intrinsically Disordered Axin-1 studied by NMR Spectroscopy and Molecular Dynamics Simulations

Rainer Bomblies[1], Manuel P. Luitz[1], Sandra Scanu[2], Tobias Madl[3,4], and Martin Zacharias[1,*]

[1] *Physik-Department, Technische Universität München, Garching, Germany*
[2] *Department Chemie, Technische Universität München, Garching, Germany*
[3] *Institute of Structural Biology Helmholtz Zentrum München, Neuherberg, Germany*
[4] *Institute of Molecular Biology & Biochemistry Center of Molecular Medicine, Medical University of Graz, Graz, Austria*
[*] *correspondence: martin.zacharias@ph.tum.de (MZ)*

Table A: Sequences of simulated Axin segments.

first residue	sequence
381	VRVEPQKFAE
391	ELIHRLEAVQ
401	RTREAEEKLE
411	ERLKRVRMEE
421	EGEDGDPSSG
431	PPGPCHKLPP
441	APAWHHFPPR
451	CVDMGCAGLR
461	DAHEENPESI
471	LDEHVQRVLR
481	TPGRQSPGPG

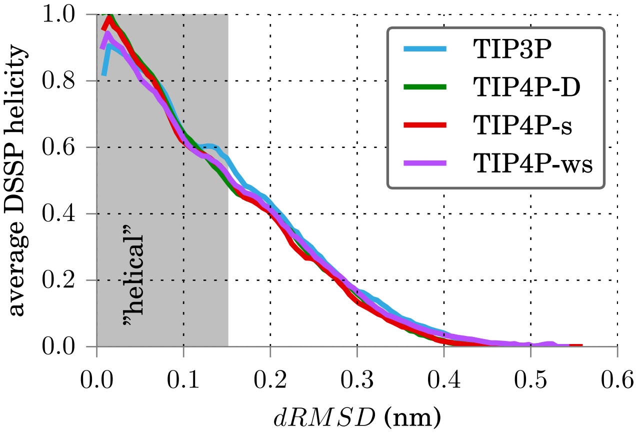

Figure A: **Helicity compared to dRMSD.** DSSP Helicity of peptides with increasing R for different water force fields. The average is taken over all segments and all replicas for each force field.

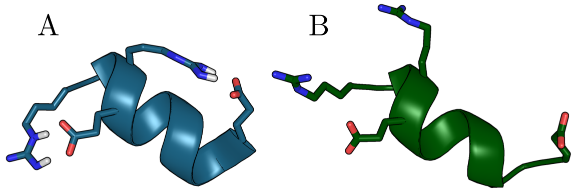

Figure B: **Snapshots of diverging typical segment conformations.** Snapshots from the lowest R replica of segment 401. A shows two stable double-H-bonds between Arg401-Glu404 and Arg403-Glu410 typically sampled with TIP3P water. B shows a typical snapshot from TIP4P-D water where neither contact is formed.

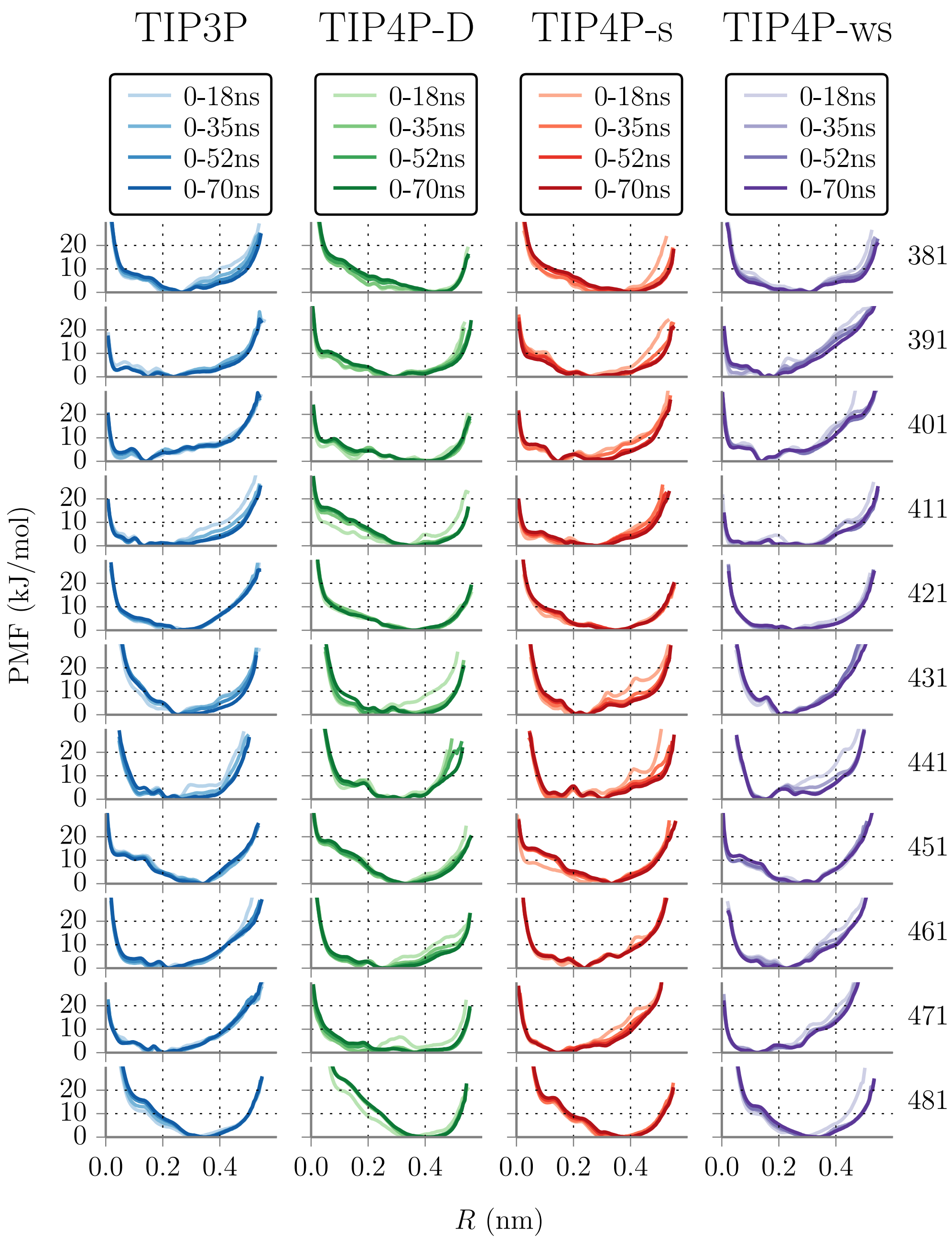

Figure C: **Time evolution of PMFs.** Time evolution of all PMFs for all water force fields. Simulation data was evaluated after 10ns of equilibration. Global shapes of the PMFs barely change after the first quarter of evaluated simulation time.

1 Implementation of the dRMSD

1.1 Distance Deviations as a Reaction Coordinate

Intrinsically disordered proteins can be in a stable conformation for microseconds. Classic continuous MD simulations can access this timescale for the desired system size and thus could capture the transition from one conformation to another. Reliable statistical averages and population probabilities, however, cannot be extracted if transitions between the states are rare. To gain any insight on the statistics of disordered proteins, advanced sampling techniques like Hamiltonian Replica exchange (H-REMD)[1] have to be used.

The crucial choice for any H-REMD method is the selection of a reaction coordinate. In the case of unfolding proteins or peptides, the coordinate should reliably distinguish between the folded state and unfolded states. The RMSD from the folded state, as used in the method of Woo & Roux[2], is a possible choice. It does, however, require a fit to the reference structure for each frame. Instead of taking the RMSD of the coordinates of atoms, here we used the RMSD of a chosen set of distances compared to respective reference distances, which can be taken from the reference structure (exemplary bonds in Figure D). This dRMSD R is defined as

$$R(d_1, \ldots, d_N) = \sqrt{\frac{1}{N} \sum_{i}^{N} (d_i - d_{i,0})^2} \tag{A}$$

Using distances avoids any fitting as the intra-molecular distances are rotation and translation invariant. In addition, the area where conformational freedom is of interest can be freely chosen by the definition of the dRMSD-pairs. With the same mechanism a single helical fragment of a protein can be unfolded or entire domains can be moved with respect to one another.

To enhance sampling along the dRMSD, harmonic potentials force the system to sample specific regions of R around a reference value R_0. The potentials with a force constant k_0 are of the form

$$V(d_1, \ldots, d_N) = \frac{1}{2} k_0 \left(R(d_1, \ldots, d_N) - R_0\right)^2 \tag{B}$$

which for pair i between atoms a and b creates a force of

$$\vec{F}_a(d_i) = -\frac{k_0}{N} \cdot \frac{R(d_i) - R_{i,0}}{R(d_i)} \cdot (d_i - d_0) \cdot \frac{\vec{r}_a - \vec{r}_b}{d_i} \tag{C}$$

pointing, depending on the sign of the R deviation, towards or away from the bond partner.

Replica exchange between Hamiltonians with different positions of the umbrella minimum further enhances sampling of different conformations and allows the system to overcome artificial barriers introduced by the additional potentials. Note that the phase space volume is not constant with respect to R.

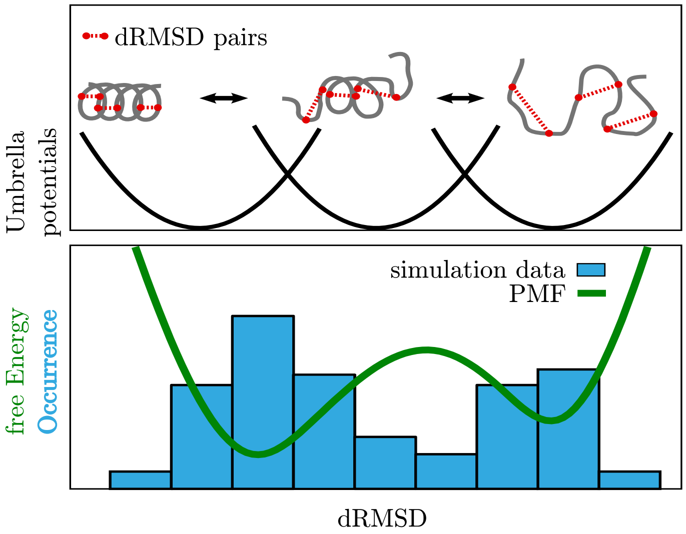

Figure D: Free energy along the dRMSD reaction coordinate. With harmonic potentials the system is forced to sample all regions of the dRMSD reaction coordinate that describes the average deviation of a chosen set of distances from a respective reference distance. The contribution of the potentials is treated by the WHAM method[3].

While, depending on the number of defined distances, only one or possibly few structures can fulfill $R = 0$, a larger R represents a vast number of conformations.

Equations of the dRMSD potential

The dRMSD is defined as

$$R(d_1, \dots, d_N) = \sqrt{\frac{1}{N}\sum_{i}^{N}(d_i - d_{i0})^2} \tag{D}$$

Where index i runs over all distances between the N atom pairs that contribute to the dRMSD.
The harmonic potentials along R are of the form

$$V(d_1, \dots, d_N) = \frac{1}{2}k_0\big(R(d_1, \dots, d_N) - R_0\big)^2 \tag{E}$$

with a specific reference dRMSD R_0 and the distance d_i being a function of the coordinates $\vec{r}_{i1}, \vec{r}_{i2}$ of the two atoms of the pair:

$$d_i \equiv d_i(r_{i1}, r_{i2}) = |\vec{r}_{i1} - \vec{r}_{i2}| = \sqrt{(\vec{r}_{i1} - \vec{r}_{i2})^2} \tag{F}$$

The forces on atom coordinate x of atom $i1$ is then calculated from

$$\begin{aligned}F_{x_{i1}} &= -\frac{dV(d_1,\ldots,d_N)}{dx_{i1}}\\ &= -\frac{k_0}{N}\cdot\frac{R(d_1,\ldots,d_N)-R_0}{R(d_1,\ldots,d_N)}\cdot(d_i-d_{i0})\cdot\frac{x_{i1}-x_{i2}}{d_i}\\ &= -F_{x_{i2}}\end{aligned}$$

Thus the vectorial force is given by

$$\vec{F}(d_i) = -\frac{k_0}{N}\cdot\frac{R(d_i)-R_0}{R(d_i)}\cdot(d_i-d_{i0})\cdot\frac{\vec{d_i}}{d_i} \tag{G}$$

Finally, the contribution to the Hamiltonian of this distance RMSD potential is

$$\mathcal{H}_{\mathrm{dRMSD}} = V(d_1,\ldots,d_N) \tag{H}$$

Lambda Scaling Along the dRMSD

For the application of US typically several windows along the reaction coordinate are defined via a transition coordinate λ. We defined the λ-dependence of R as

$$R(d_i,\lambda) = \sqrt{\frac{1}{N}\sum_i^N\left(d_i-(1-\lambda)d_{i0}^A-\lambda d_{i0}^B\right)^2} \tag{I}$$

The λ-dependent distance RMSD potential has then the form

$$V(d_i,\lambda) = \frac{k_0}{2}\left(R(d_i,\lambda)-(1-\lambda)R_0^A-\lambda R_0^B\right)^2 \tag{J}$$

This allows transitions from one state with reference distances d_{i0}^A to another state with reference distances d_{i0}^B. Also, with no d_{i0}^B defined, a continuous sampling of λ in the range $[0,1]$ allows sampling from the structure defined with distances d_{i0}^A to unfolded structures up to a dRMSD deviation of R_0^A.

The derivative of the potential with respect to λ then is

$$\begin{aligned}\frac{dV(d_i,\lambda)}{d\lambda} =& k_0\left(R(d_i,\lambda)-(1-\lambda)R_0^A-\lambda R_0^B\right)\cdot\\ &\left(\frac{dR(d_i,\lambda)}{d\lambda}+R_0^A-R_0^B\right)\\ =& k_0\left(R(d_i,\lambda)-(1-\lambda)R_0^A-\lambda R_0^B\right)\cdot\\ &\left(\frac{1}{2NR(d_i,\lambda)}\left(2\sum_i^N\left(d_i-(1-\lambda)\,d_{i0}^A-\lambda d_{i0}^B\right)\left(d_{i0}^A-d_{i0}^B\right)\right)+\right.\\ &\left.R_0^A-R_0^B\right)\end{aligned} \tag{K}$$

and the force in direction x for atom $i1$ is

$$\begin{aligned} F_{x_{i1}} =& -\frac{dV(d_i,\lambda)}{dx_{i1}} \\ =& -\frac{k_0}{N}\cdot\frac{R(d_i,\lambda)-(1-\lambda)R_0^A-\lambda R_0^B}{R(d_i,\lambda)}\cdot \\ & \left(d_i-(1-\lambda)d_{i0}^A-\lambda d_{i0}^B\right)\cdot\frac{x_{i1}-x_{i2}}{d_i} \\ =& -F(x_{i2}) \end{aligned} \tag{L}$$

and thus the vectorial force is

$$\begin{aligned} \vec{F}(d_i,\lambda) =& -\frac{k_0}{N}\cdot\frac{R(d_i,\lambda)-(1-\lambda)R_0^A-\lambda R_0^B}{R(d_i,\lambda)}\cdot \\ & \left(d_i-(1-\lambda)d_{i0}^A-\lambda d_{i0}^B\right)\cdot\frac{\vec{d_i}}{d_i} \end{aligned} \tag{M}$$

Gromacs Implementation

The dRMSD as a reaction coordinate for umbrella sampling with replica exchange has been implemented in GROMACS[4] 4.6.2 and uploaded to github: `https://github.com/enzyx/gromacs-4.6-drmsd`
To run a simulation with the adapted GROMACS code the following settings are required.

MDP Parameters

`drmsd-pot:  yes/no`
  Enable/Disable distance based RMSD potential.

`drmsd-ref:  (0.0) [nm]`
  Reference distance RMSD for state A

`drmsd-refB: (0.0) [nm]`
  Reference distance RMSD for state B

`drmsd-k0:  (1000.0) [kJ mol`$^{-1}$ `nm`$^{-2}$`]`
  Force konstant of the distance RMSD potential

`nstdrmsdpout:  (100)`
  Frequency of writing distance RMSD potential output

`bonded-lambdas = 0.00 0.20 0.40 0.60 0.80 1.00`
  λ values for umbrella windows. (requires further free energy parameters)

Topology File

The topology needs to include a `[ drmsd_restraints ]` section:

```
Topology file parameters:
[ drmsd_restraints ]
;   ai      aj      type    d0      d0B (optional)
    9       39      1       0.5     0.6
    19      49      1       0.5     0.6
    29      59      1       0.5     0.6
```

Atom pair `ai`, `aj` which forms a distance pair. Default function type 1. `d0` is the reference distance of this atom pair. Note that `ai`, `aj` are molecule interal indices. The first atom of each molecule has index 1.

mdrun Parameters

The possible long distance bonds that have to be calculated each step clash with the domain decomposition principle of Gromacs. Simulations will not start without the explicit request of particle decomposition:

```
mdrun [...] -pd
```

g_drmsd

The implementation of the distance restraint can write out the dRMSD calculated during the simulation. Additionally the tool `g_drmsd` can be used to calculate dRMSDs from a given trajectory. To obtain the distances and applied forces use `g_drmsd`. `g_drmsd` has to be given at trajectory and a run input file with all the settings for the dRMSD method. The tool then for each frame of the trajectory extracts the dRMSD and the resulting potential to a output file.

```
-f Input, trajectory: .xtc, .trr etc.
-s Input, run input file: .tpr
-o Output file (drmsd.xvg), optional
```

If `g_drmsd` is given a list of trajectories and tpr files it will calculate the drmsd and potential for the first given trajectory with the first tpr and so forth. Non-matching numbers after the last underscore, e.g. traj_1.xtc and topol_2.tpr will give an error. Output for each trajectory will be written to files with matching number.

References

[1] Manuel Luitz, Rainer Bomblies, Katja Ostermeir, and Martin Zacharias. Exploring biomolecular dynamics and interactions using advanced sampling methods. *Journal of Physics: Condensed Matter*, 27(32):323101, 2015.

[2] Hyung-June Woo and Benoit Roux. Calculation of absolute protein–ligand binding free energy from computer simulations. *Proc. Natl. Acad. Sci. U.S.A.*, 102(19):6825–6830, 2005.

[3] Shankar Kumar, John M. Rosenberg, Djamal Bouzida, Robert H. Swendsen, and Peter A. Kollman. The weighted histogram analysis method for free-energy calculations on biomolecules. i. the method. *Journal of Computational Chemistry*, 13(8):1011–1021, 1992.

[4] Sander Pronk, Szilárd Páll, Roland Schulz, Per Larsson, Pär Bjelkmar, Rossen Apostolov, Michael R. Shirts, Jeremy C. Smith, Peter M. Kasson, David van der Spoel, Berk Hess, and Erik Lindahl. Gromacs 4.5: A high-throughput and highly parallel open source molecular simulation toolkit. *Bioinformatics*, 29(7):845–854, 2013.
